# Advanced PMSSO Hydrogel Cross-Linked Cyclodextrin Composite Carrier for Enhanced Oral Delivery of Iron to Treat Anemia

**DOI:** 10.3390/gels11120973

**Published:** 2025-12-02

**Authors:** Polina Orlova, Sergei Sharikov, Vsevolod Frolov, Alexey Doroshenko, Ivan Meshkov, Anna Skuredina, Grigorii Lakienko, Egor Latipov, Alexandra Kalinina, Aziz Muzafarov, Irina Le-Deygen

**Affiliations:** 1 Department of Chemistry, Lomonosov Moscow State University, Moscow 119991, Russia; p.orlova2021@mail.ru (P.O.); sharikovsv@my.msu.ru (S.S.); frolov_vsevolod@mail.ru (V.F.); alexey07251@gmail.com (A.D.); skuredinanna@gmail.com (A.S.); bojk25@gmail.com (G.L.); 2 Enikolopov Institute of Synthetic Polymeric Materials, Russian Academy of Sciences (ISPM RAS), Moscow 117393, Russia; ivanbm@ispm.ru (I.M.); kalinina@ispm.ru (A.K.); aziz@ispm.ru (A.M.); 3 Institute of Nanotechnology of Microelectronics, Russian Academy of Sciences (INME RAS), Moscow 115487, Russia; la_019@mail.ru

**Keywords:** iron deficiency anemia, organosilicon hydrogels, cyclodextrins, drug delivery

## Abstract

Iron deficiency anemia continues to pose a significant global health burden, necessitating the development of improved therapeutic delivery systems. This study investigates novel composite materials composed of organosilicon hydrogels and cross-linked sulfobutyl ether beta-cyclodextrin (SBECD) nanoparticles for the oral delivery of iron compounds. Two types of cross-linked SBECD nanoparticles were synthesized using 1,6-hexamethylene diisocyanate. These nanoparticles were characterized by DLS, NTA, and FTIR and possess size around 200–300 nm and negative zeta-potential around −35 mV with molecular weight 150–200 kDa. Various hydrogel matrices, including plain PMSSO hydrogels and modified versions with amino groups or silicate cross-links, are also described. The hydrogels were evaluated for their iron sorption capacity (up to 44% loading efficiency) and release kinetics for 3 h. The results demonstrate that cross-linked SBECD nanoparticles significantly enhance iron sorption and provide sustained release under simulated physiological conditions. Mathematical modeling indicated that the Higuchi model best describes the iron release kinetics. The findings suggest that the proposed composite materials hold considerable promise for the treatment of iron deficiency anemia, offering an innovative approach to enhance therapeutic efficacy and minimize adverse effects.

## 1. Introduction

Iron deficiency anemia poses a significant global health challenge, impacting individuals across all age groups [1]. An inadequate supply of iron within the body triggers a cascade of debilitating symptoms that profoundly compromise the overall quality of life for affected patients. Current therapeutic strategies hinge critically upon selecting appropriate modes of drug administration. In emergent cases where immediate intervention is required, clinicians often opt for intravenous infusion. However, this route comes at the cost of potential adverse reactions affecting vital organs such as the hematopoietic system and kidneys.

In contrast, oral formulations represent the mainstay for chronic management of anemic syndrome. These regimens enable ambulatory care but are marred by undesirable gastrointestinal disturbances. It is reported that about half of patients suffer from such side effects [2]. Consequently, there exists an urgent need to develop novel drug delivery platforms capable of circumventing these limitations.

Recent advances in material science offer promising alternatives aimed at enhancing bioavailability and minimizing off-target toxicity. Specifically, biocompatible polymers, including alginate-based matrices [3], and advanced nanostructures, such as liposomal carriers [4], demonstrate great promise in optimizing controlled-release profiles. Furthermore, innovative technologies, exemplified by microneedle-mediated transdermal delivery [5], provide exciting opportunities for site-specific targeting.

Our research group has pioneered an alternative strategy centered on organosilicon poly(methyl silsesquioxane) (PMSSO)-based hydrogel systems. This unique platform demonstrates exceptional capacity for binding and sustained release of iron ions under simulated physiological conditions [6]. Moreover, we have explored synergistic benefits arising from co-formulation with β-cyclodextrin derivatives, leading to further refinement of release kinetics [7]. Further, composite materials based on organosilicon hydrogels have potential for biomedical applications [8].

The ongoing optimization efforts seek to refine the release kinetics of iron-containing therapeutics encapsulated within carriers. Cyclodextrins cross-linking generates nano- and submicron-sized particles [9]. These structures serve as versatile carriers, facilitating host–guest interactions between themselves and entrapped molecules, thereby influencing the kinetics of drug release [10]. Previous studies have illustrated analogous outcomes when employing low molecular weight antibiotic complexes [11]. The possibility of regulating the kinetics of cargo release from hydrogels through complex formation with cyclodextrins was reported by Quaglia [12].

Composite materials based on hydrogels and cyclodextrins have attracted the attention of many researchers and have already proven their worth. A recent review [13] provides a comprehensive description of such systems, in which a drug is incorporated into a cyclodextrin complex, and the complex itself is loaded into a hydrogel. The combination of a polymer as a matrix carrier and cyclodextrins as complexing agents for the active molecule has previously demonstrated its potential [14]. However, the question of using cross-linked cyclodextrin particles for such composites remains open: both from the point of view of the fundamental properties of such composites and from the applied point of view for the creation of drug delivery systems, especially considering cross-linked cyclodextrin as a carrier by itself [15].

Building upon these findings, our current investigation aims to synthesize hybrid formulations consisting of cyclodextrin-derived nanoparticles loaded with ferrous D-gluconate (Fe(II)) as a model iron compound. Subsequent incorporation into PMSSO hydrogels represents a strategic step toward designing enhanced oral therapies specifically tailored for managing iron deficiency anemia.

## 2. Results and Discussion

In this study, we designed the following nanocomposite system: iron(II) D-gluconate loaded into cross-linked cyclodextrin nanoparticles. This complex is embedded within structurally varied poly(methyl silsesquioxane) (PMSSO) hydrogels. Our objective was twofold: first, to investigate the interplay between structural modifications of the hydrogel matrix, structure of cross-linked cyclodextrin nanoparticles and physico-chemical properties of final nanocomposite; second, to tailor release profile and disclose patterns of Fe and sulfobutyl ether beta-cyclodextrin (SBECD) derivatives release.

### 2.1. Synthesis and Characterization of Cross-Linked Cyclodextrin Nanoparticles

We aimed to synthetize nanoparticles based on the cross-linked β-cyclodextrin as previously preferable release profiles were demonstrated for other low-molecular drugs [11]. Here, we have chosen as a monomer sulfobutyl ether beta-cyclodextrin, with cavity of optimal diameter of approximately 0.60–0.65 nanometers and a height of about 0.78 nanometers. It is known that the charged sulfobutyl ether groups do not dramatically change the cavity size for drug loading but could beneficially influence on solubility, complexation ability, and molecular interactions [16]. We have used the torus cross-linking strategy to form a dense mesh as described in [17].

**Scheme 1:**
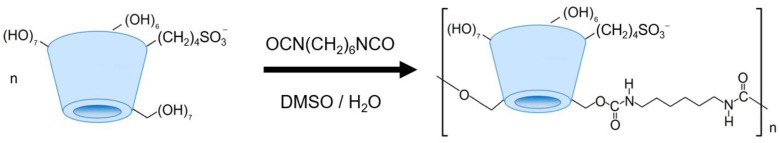
The scheme of cross-linked sulfobutyl ether beta-cyclodextrin synthesis.

The dense of cross-linking could play a dramatic role in the sorption capacity [10]. Thus, we have synthetized two types of particles, achieved through covalent cross-linking of individual cyclodextrin units with varied amount of 1,6-hexamethylene diisocyanate (HMDI) at the hydroxyl moieties of the SBECD molecules. This process was carried out in a water–dimethylsulfoxide (DMSO) mixture. As depicted schematically in Scheme 1, the synthesis involved primarily the more reactive primary hydroxyl groups of the cyclodextrin monomers. Nonetheless, secondary hydroxyl groups could also engage in the reaction, contributing to the formation of a branched macromolecular architecture.

Particles labeled as Pol-SBECD-1 were synthetized via 0.5 molar excess of HMDI, while Pol-SBECD-2 required equimolar amount of HMDI. Adjustments in HMDI concentration were strategically implemented to yield particles characterized by differential degrees of cross-linking density. Characterization techniques included dynamic light scattering (DLS), nanoparticle tracking analysis (NTA), and Fourier-transform infrared spectroscopy (FTIR) in attenuated total reflection (ATR) mode. Key parameters of the resultant particles are summarized in Table 1.

Using a greater excess of cross-linking agent promotes the formation of smaller particles with a lower degree of polymerization. This is likely due to the formation of denser cross-links in the particles, while small amounts of HMDI promote the formation of “loose” particles with a larger hydrodynamic radius and a greater number of cross-linked tori. Moreover, despite significant differences in the degree of polymerization, the zeta-potential of the particles remains comparable, probably due to the contribution of only the surface tori. A similar result was reported by Gabr for cross-linked particles of carboxylated cyclodextrin [18].

**Figure 1:**
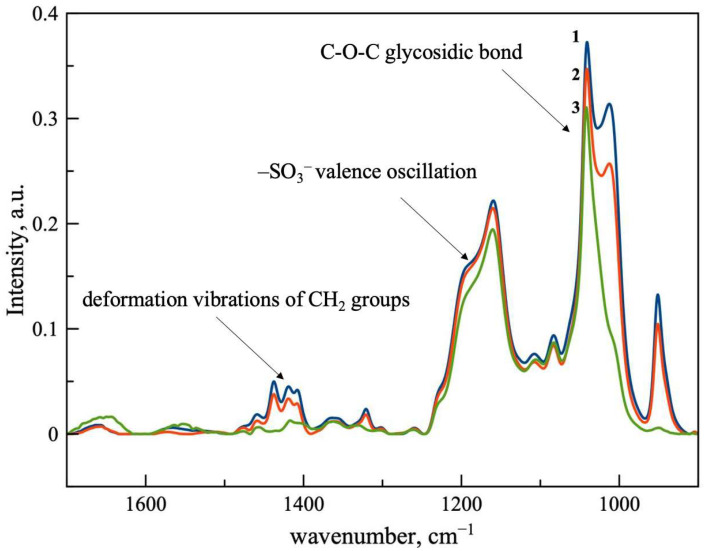
ATR-FTIR spectra of SBECD (3, green line) and cross-linked particles Pol-SBECD-1 (1, blue line) and Pol-SBECD-2 (2, red line). Concentration of cyclodextrin tori is 0.1 M in all samples. Aqueous solutions at 22 °C.

Figure 1 shows the ATR-FTIR spectra of the synthesized particles compared to the spectrum of the original SBECD. Here, we present the most informative area of the spectra, while full-view spectra are available on Figure S1. This method has proven effective for the characterization of cross-linked materials [19,20]. In the FTIR spectrum of the β-CDs, analytically significant absorption bands are in the wavenumber range of 1250–900 cm^−1^. The most intense absorption band at 1100–1000 cm^−1^ corresponds to the oscillations of the C-O-C glycosidic bond, and the wider band in the range of 1250–1190 cm^−1^ corresponds to vibrations of the –SO_3_^−^ groups. The band at 1040 cm^−1^ is the most intense in the spectrum and appears to be convenient for analyzing the content of CD’s tori, both in solutions of the original SBECD and in cross-linked particles. Cross-linking leads to the formation of a network of CD tori, as indicated by the appearance of a shoulder at 1013 cm^−1^, as well as an increase in the shoulder at 1195 cm^−1^. We also observed the appearance of a new absorption band at 951 cm^−1^, corresponding to vibrations of the C-N bond. The combination of bands in the region of 1400–1500 cm^−1^ corresponds to the deformation vibrations of CH_2_ groups in the cross-link. It has been previously shown that such changes in spectra are typical for cross-linking of cyclodextrins [21]. Thus, we obtained cross-linked SBECD particles with negative zeta-potential that could be favorable for iron sorption.

### 2.2. Patterns of Sorption of Iron Gluconate by Derivatives of SBECD

**Scheme 2:**
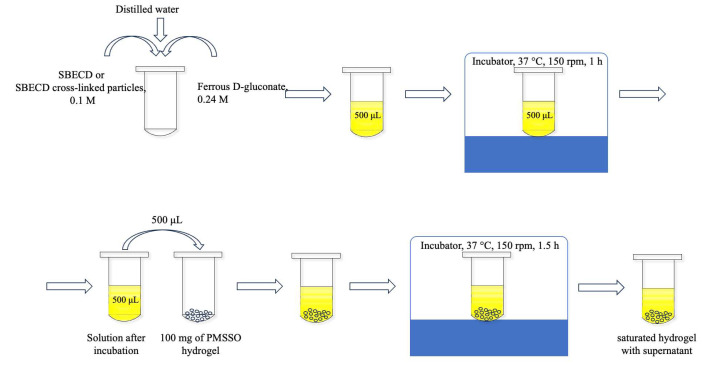
Schematic diagram of the production of a composite ferrous D-gluconate delivery system based on beta-cyclodextrin derivatives and PMSSO hydrogels.

We obtained complexes, as is represented in Scheme 2. Complexation between SBECD or synthetized particles and ferrous D-gluconate was studied using ATR-FTIR, owing to the distinct spectra of both compounds.

**Figure 2:**
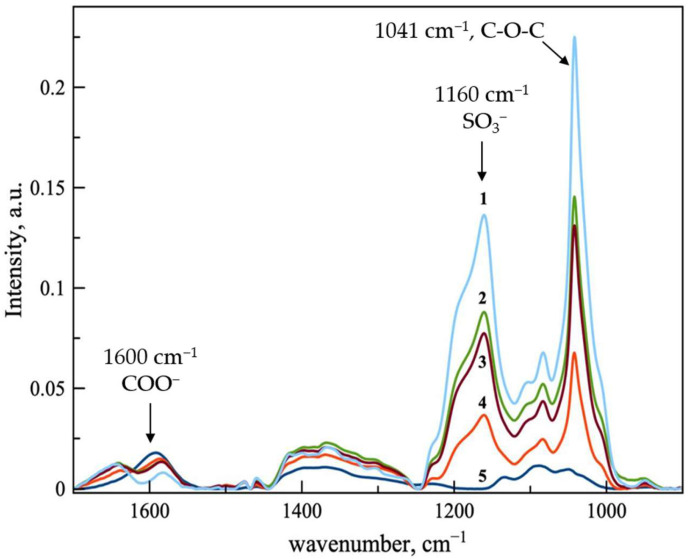
ATR-FTIR spectra of ferrous D-gluconate (line 5, blue) and ferrous D-gluconate complexes with SBECD in variable molar ratios: line 1—1:2; 2—1:1.5; 3—1:1; 4—2:1. Aqueous solutions at 22 °C.

Ferrous D-gluconate and SBECD complexes were obtained in various ferrous D-gluconate–SBECD molar ratios of 2:1, 1:1, 1:1.5, and 1:2. The IR spectra of the resulting samples are shown in Figure 2.

The obtained data indicate that, with the increase in CD’s concentration, the intensities of the characteristic bands associated with SBECD raise consistently (in particular, the bands at 1159 and 1041 cm^−1^, corresponding to vibrations of the –SO_3_ groups and the C-O-C glycosidic bond). At the same time, we noticed a decrease in the intensity of the 1601 cm^−1^ band, characteristic of the asymmetric vibrations of COO^−^ in iron gluconate. It is assumed that the formation of the most stable complex occurs at a twofold excess of CD, when the complete incorporation of gluconate molecules into the CD cavities is most likely possible.

For sorption characteristics of cross-linked SBECD, we obtained complexes of the particles with ferrous D-gluconate at various ratios of ferrous D-gluconate–SBECD torus (2:1, 1:1, and 1:1.5). Torus concentration was controlled with ATR-FTIR by means of calibration curve for the band 1041 cm^−1^.

**Figure 3:**
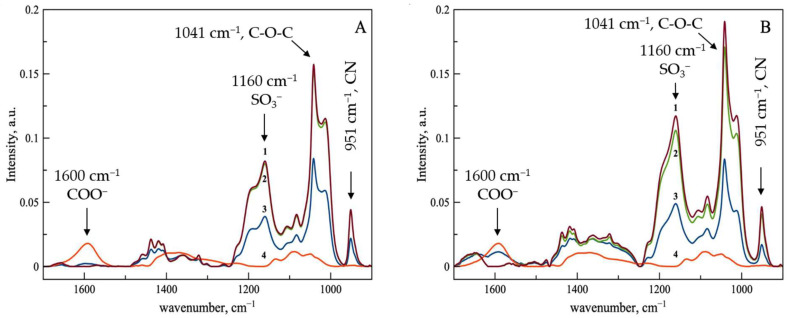
ATR-FTIR spectra of ferrous D-gluconate (line 4, red) and its complexes with cross-linked SBECD particles in various molar ratio (line 1, brown—1:1.5, line 2, green 1:1, line 3, blue—2:1). Figure (**A**) represents data for Pol-SBECD-1, and (**B**) is for Pol-SBECD-2. Aqueous solutions at 22 °C.

Figure 3A illustrates the ATR-FTIR spectral data for the complexes formed between Pol-SBECD-1 and ferrous D-gluconate. Upon detailed examination of the spectra, it becomes evident that the intensities of key spectral bands increase proportionately with rising concentrations of SBECD tori incorporated into the particles. Strikingly, a more precipitous decline in the intensity of the asymmetric carboxylate stretching vibration band at 1601 cm^−1^ is discernible relative to the adsorption spectrum of the original SBECD. This observation strongly suggests that the cross-linking chemistry plays a pivotal role in modulating the coordination environment of the ferrous ion. Additionally, a marked reduction in the peak intensity at 1364 cm^−1^, attributable to the hydroxyl stretch of D-gluconate, emerges prominently in comparison to the baseline absorbance pattern of pure SBECD. Collectively, these trends corroborate the hypothesis that cross-linking facilitates enhanced stabilization of the metal-ligand interaction within the cyclodextrin lattice.

As presented in Figure 3B, the ATR-FTIR spectra for the complexes comprising Pol-SBECD-2 and ferrous D-gluconate reveal a parallel enhancement in band intensities concomitant with augmented SBECD tori loadings. Despite this general agreement, subtle differences emerge regarding the comparative visibility of characteristic gluconate-related absorptions. More specifically, the intensity of these features appears amplified vis-à-vis the preceding dataset. Such discrepancies plausibly arise from reduced efficiencies in accommodating gluconate within the confined interior spaces of the cyclodextrin cavities. The heightened rigidity imparted by the underlying polymer scaffolding—presumably manifesting through hindered conformational flexibility—may impede full engagement of the ligand with the macrocyclic host, yielding lower affinities for coordinated binding.

**Figure 4:**
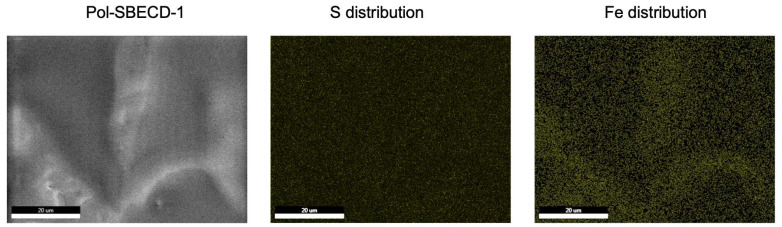
Scanning electron microscopy and EDX for the complex Pol-SBECD-1—ferrous D-gluconate.

The above-described findings are supported by scanning electron microscopy (SEM) and energy-dispersive X-ray spectroscopy (EDX) analyses. Figure 4 depicts representative micrographs illustrating the morphological features of Pol-SBECD-1 particles loaded with ferrous D-gluconate. Elemental maps highlighting sulfur atoms demonstrate the even distribution of CD tori, along with the homogenous spatial arrangement of iron atoms. Remarkably, no discernible areas devoid of iron enrichment are detectable, suggesting uniformity in iron distribution in the carrier.

Consequently, cross-linking processes exert a decisive influence on the sorption properties of SBECD derivatives. The cross-linked polymer configuration exhibits notably enhanced complexation efficiency relative to its native counterpart. Conversely, however, the magnitude of cross-linking exerts a proportional impact on the overall sorption behavior. With increased utilization of hexamethylene diisocyanate, the number of available active sites for engaging with gluconate gradually diminishes. This phenomenon translates into progressively less pronounced alterations in spectral signatures, mirroring the evolving structural integrity and polymerization degree of the particles.

Henceforth, SBECD particles were successfully produced and have proved remarkable sorption capabilities for ferrous D-gluconate. Moving forward, the next step involves embedding these particles within organosilicon hydrogels. Given the importance of choosing an appropriate hydrogel architecture to ensure maximal particle loading and optimal release profiles, we synthesized several variants of hydrogels, each embodying distinct structural arrangement.

### 2.3. PMSSO-Hydrogels Synthesis and Characterization

We synthesized PMSSO hydrogels of various structures (Scheme 2). As a control, we studied a typical PMSSO hydrogel with silsesquioxane structure only, while other samples possess several modifications: either extra MeSiO_1.5_/SiO_2_ links or extra amino groups. We hypothesized that presence of amino groups in the polymer structure will support complex formation with charged SBECD cross-linked particles, while additional cross-linking could influence drug loading kinetics.

**Scheme 3:**
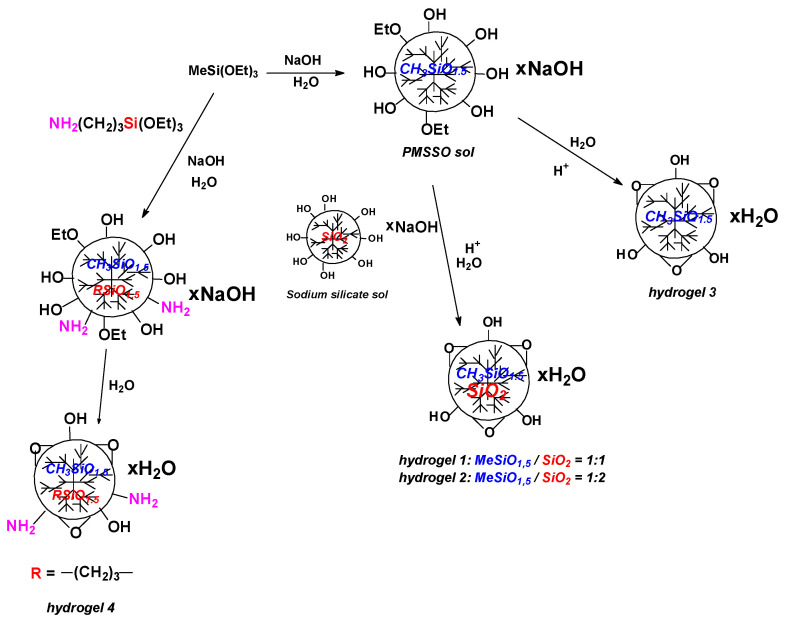
Synthesis of PMSSO hydrogels with variable structure.

Poly(methyl silsesquioxane) (PMSSO) hydrogels of varying architectures were prepared, as outlined in Scheme 3. A standard PMSSO hydrogel with solely silsesquioxane structure served as a control, while other samples featured several modifications, including the introduction of MeSiO_1.5_/SiO_2_ linkages or terminal amino functionalities. Our working hypothesis posited that the incorporation of amino groups within the polymer backbone would facilitate stable complexation with charged SBECD cross-linked particles. Simultaneously, we anticipated that incremental cross-linking modifications would influence the kinetics of drug loading and subsequent release behaviors.

The hydrogels synthesized herein exhibit significant surface areas, as quantified by Brunauer–Emmett–Teller (BET) measurements (Table 2). Amongst the evaluated materials, the PMSSO hydrogel composed exclusively of silsesquioxane motifs displayed the highest specific surface area. This reproducible high [22] specific surface area is an advantage of the original PMSSO hydrogel. Optimization of the synthesis parameters allow for obtaining PMSSO xerogels with a specific surface area about 600 m^2^/g. Other hydrogels with high specific surface area are also known [23]. The authors emphasize the importance of synthesis conditions to achieve values above 600. By contrast, other hydrogels synthesized in this work exhibited specific surface areas at least halved in magnitude.

Despite this disparity, when scrutinizing the sorption capacity for the model dye Congo red, a striking augmentation in uptake capability emerged for the amine-functionalized variant of the hydrogel.

**Figure 5:**
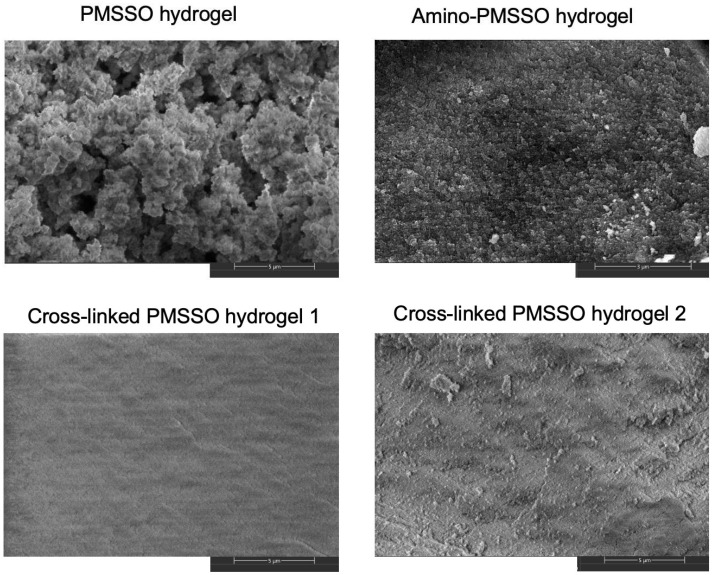
Scanning electron microscopy of PMSSO hydrogel, amino-PMSSO hydrogel, and cross-linked hydrogels with various MeSiO_1.5_/SiO_2_ ratio.

The morphological characteristics of the synthesized hydrogels were comprehensively assessed via scanning electron microscopy (SEM), as illustrated in Figure 5. Additional micrographs for the samples are available in Figures S2–S5. Observations revealed that the pristine PMSSO hydrogel possesses a highly developed surface area accompanied by a distinctive “curly” texture. In contrast, the aminated derivative exhibits a considerably finer granular dimensionality. Introducing additional silicate cross-links dramatically transforms the morphology of the samples, supplanting the intricate surface topology with a smoother structure punctuated by discrete features. This observation is in good agreement with the BET data: the original PMSSO hydrogel indeed has the most developed surface. Therefore, despite retaining comparable chemical constitutions, the presence of cross-links within PMSSO hydrogels exerts a profound influence on their overall morphology and attendant physicochemical properties.

An imperative consideration pertains to understanding how hydrogel architecture influences sorption patterns. Specifically, it became necessary to evaluate the sorption capacities of the synthesized hydrogels concerning both ferrous D-gluconate and its complexes with SBECD derivatives. Assessing these factors enables the identification of optimal formulations capable of supporting the desired release profiles.

### 2.4. Patterns of Sorption of Ferrous D-Gluconate Complexes with SBECD in PMSSO Hydrogels

Does the molecular architecture of the PMSSO hydrogel dictate its sorption properties for ferrous D-gluconate and its complexes with cross-linked particles? To address this inquiry, we conducted a comparative assessment of iron loading efficiency, normalized per unit mass of pure iron, focusing explicitly on hydrogels bearing amino functionalities versus those lacking additional modifications. The compiled results are presented in Table 3.

**Figure 6:**
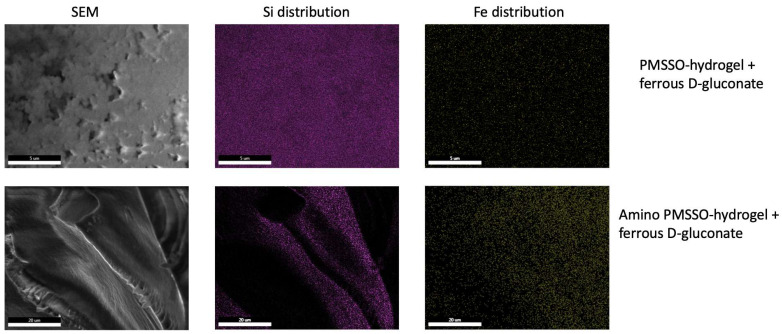
Scanning electron microscopy and EDX (Si and Fe) for the PMSSO hydrogel and amino-PMSSO hydrogel loaded with ferrous D-gluconate.

Upon comparing the loading efficiency of hydrogels directly loaded with ferrous D-gluconate, it was found that the presence of amino groups does not confer a substantial advantage in terms of Fe uptake. These findings are validated by scanning electron microscopy and energy-dispersive X-ray spectroscopy data (Figure 6), which illustrate the colocalization of iron and silicon components within hydrogel samples.

To load ferrous D-gluconate complexes with SBECD derivatives into hydrogels, long-term incubation with stirring was used, after which the hydrogels were separated from the solution and the loading efficiency was assessed. Formation of complexes between ferrous D-gluconate and cross-linked SBECD significantly enhances the efficiency of iron loading into hydrogels. Notably, the employment of Pol-SBECD-1 particles consistently boosts loading efficiency across both gel varieties, whereas the combination of Pol-SBECD-2 particles with ferrous D-gluconate in an amino-PMSSO hydrogel achieves the most favorable loading outcomes.

**Figure 7:**
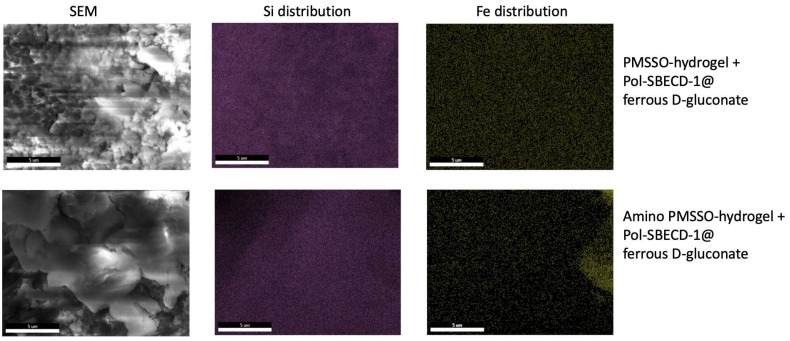
Scanning electron microscopy and EDX (Si and Fe) for the PMSSO hydrogel and amino-PMSSO hydrogel loaded with complex Pol-SBECD-1@ferrous D-gluconate.

To gain further insights, the morphology of loaded hydrogels was investigated using SEM and EDX to monitor the distribution of iron and silicon (Figure 7). Examination revealed a well-developed morphology, characterized by the co-localization of iron and silicon elements, thus reinforcing the inferred loading efficiency.

Considering cross-linked hydrogels, we observed insufficient sorption efficiency for iron complexes with SBECD derivatives. Given the surface morphology, this was expected—additional cross-linking likely hinders the effective sorption of large cross-linked SBECD particles. Thus, unmodified PMSSO hydrogel and amino-PMSSO were selected for further release kinetic studies.

### 2.5. Release Kinetics

The kinetics of cargo release from hydrogels depends on many factors and can be described by a variety of models [24]. For studying the release kinetics, the following combinations were chosen: PMSSO hydrogel and amino-PMSSO hydrogel served as matrices, while ferrous D-gluconate and its complexes with either simple SBECD or cross-linked SBECD particles (both Pol-SBECD-1 and Pol-SBECD-2 types) acted as payloads. In these systems, the precise release mechanism remains ambiguous: is iron released purely from the hydrogel, or does it remain partially associated with CD derivatives during the release process? To what extent does complex formation influence the iron release?

**Figure 8:**
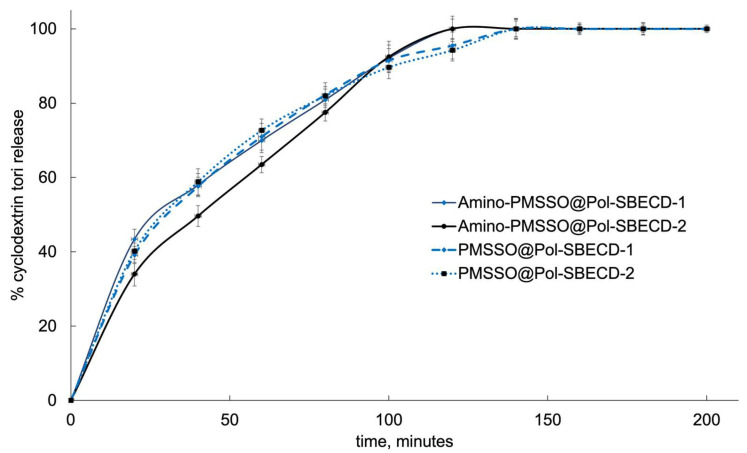
Kinetics of cyclodextrin tori release from different samples. The first 120 min of the experiment corresponds to the gastric media, with pH 1.4, followed by 80 min in the intestinal medium, with pH 7.4 at 37 °C. SD, *n* = 3.

To clarify these issues, we conducted control experiments examining the kinetic profiles of “empty” SBECD cross-linked particles loaded into PMSSO-hydrogels (Figure 8). All obtained release curves were similar, revealing negligible deviations among the different particle and/or hydrogel samples. Within the first two hours, the complete particle release occurred at pH 7.4. Interestingly, a delay in release was observed for the system combining amino-PMSSO with Pol-SBECD-2 particles, hinting at possible electrostatic interactions between the hydrogel’s amino moieties and the sulfonate groups present in the particles.

To precisely capture the behavior of the studied systems, the kinetic curves were analyzed within the context of established mathematical frameworks [25]. Several plausible models exist for describing drug release mechanisms in pharmaceutical delivery systems. The zero-order model is characterized by constant release rates irrespective of drug concentration, typically applicable to slow-solubility matrices. First-order kinetics is relevant for porous matrices, where drug release depends linearly on the residual drug concentration. However, considering that diffusion mechanisms play a dominant role in many practical scenarios, more sophisticated models such as the Higuchi or Korsmeyer–Peppas equations are frequently applied. Table 4 summarizes the coefficients of determination (R^2^) derived from fitting the kinetic data of cyclodextrin particle release. For samples without ferrous D-gluconate, the Higuchi model yields the highest R^2^, closely followed by the Korsmeyer–Peppas model. Since the latter was originally formulated for polymeric matrices like hydrogels [26,27], we propose that this model offers the greatest relevance.

**Figure 9:**
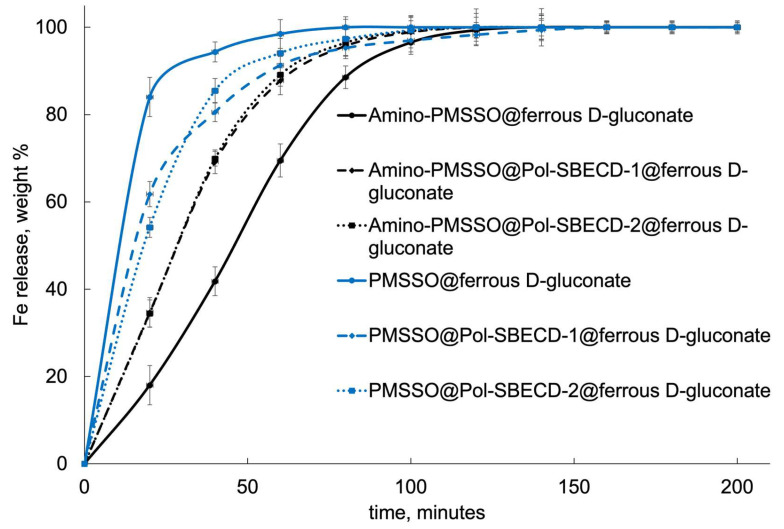
Fe release curves from the obtained systems. The first 120 min of the experiment corresponds to the gastric media, with pH 1.4, followed by 80 min in the intestinal medium, with pH 7.4 at 37 °C. SD, *n* = 3.

Figure 9 presents data on the kinetic curves depicting iron release dynamics. Unmodified PMSSO hydrogels display the swiftest rate of iron release, completing nearly total release within just under an hour. In contrast, the aminated PMSSO hydrogel demonstrates a conspicuous lag phase in the acidic media, indicating prolonged retention of iron.

Introducing complexes with cross-linked particles significantly alters this scenario. In the case of the aminated hydrogel, iron release accelerates noticeably, with no distinguishable variation between the two types of particles employed. On the contrary, in the unmodified hydrogel, the incorporation of particles induces a retardation effect, decelerating the release kinetics.

Figure 9 demonstrates the kinetic curves for iron release. The unmodified PMSSO hydrogel exhibits the fastest iron release: it takes less than an hour for almost complete release. In contrast, the amino-PMSSO hydrogel exhibits a clearly delayed release in the acidic environment. The use of complexes with cross-linked particles changes the picture: for the aminated hydrogel, release is accelerated, with no clear differences observed between the two particle types. Conversely, for the unmodified gel, the use of particles allows for a slower release.

Comparing to the previously published data, it was demonstrated that complex formation between cyclodextrin and iron ions provides controlled dissociation and protects iron from premature release and degradation in acidic environments [28].

Which model best captures the kinetics of iron release? Table 5 summarizes the coefficient of determination (R^2^) values obtained from the mathematical analysis of the experimental curves. The results unequivocally favor the Higuchi model, as previously established in our related work investigating analogous systems [7]. Moreover, close results were obtained by Machín et al. [29], where drug release kinetics followed a Fickian diffusion mechanism and were fitted with the Korsmeyer–Peppas and Higuchi models.

**Figure 10:**
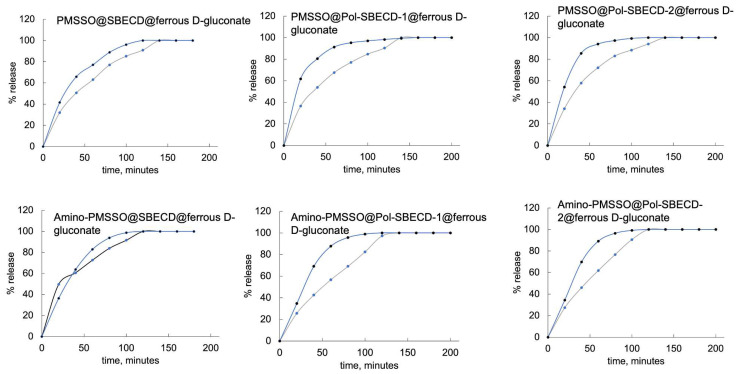
Kinetics of iron (blue line) and cyclodextrin tori (gray line) release. First 120 min of the experiment corresponds to the gastric media, with pH 1.4, followed by 80 min in the intestinal medium, with pH 7.4 at 37 °C.

Is the release of particles linked synchronously with the release of iron, or does it proceed sequentially? We compared the release kinetics of iron and CD particles. Figure 10 visually contrasts the release profiles for each component within the respective systems. Notably, the concurrent release of iron and non–cross-linked SBECD from the hydrogel matrix implies limited retention strength of the complex within the gel. Across all examined systems, the temporal evolution of release trajectories diverges, indicating that iron is liberated ahead of the particles. Most probably, the release commences from the superficial layers of the hydrogel. Here, particles appear to linger longer within the matrix, subsequently giving rise to a synchronized dual-phase release process encompassing both the intact Pol-SBECD-ferrous D-gluconate complex and isolated ferrous D-gluconate molecules.

The obtained data are in good agreement with those previously reported in the literature. For example, Machin [29] showed that, for a hydrogel consisting of cyclodextrin cross-linked with epichlorohydrin, the release kinetics of antifungal drugs also obeys the Korsmeyer–Peppas equation.

## 3. Conclusions

In this study, we developed a novel composite material leveraging organosilicon hydrogels with tunable architectures and cyclodextrin derivatives for the delivery of iron compounds, exemplified by ferrous D-gluconate. The synthesized cross-linked SBECD particles exhibited remarkable iron sorption capabilities, with iron evenly dispersed throughout the particles, as verified by the SEM and EDX analyses.

To construct the hydrogel matrix, we synthesized both basic PMSSO hydrogels and variants enriched with silicate cross-links or amino functionalities. Structural modifications induced profound changes in hydrogel morphology, as evidenced by the SEM examinations, and exerted a measurable influence on their sorption capacities for iron-bearing compounds. Both the control and aminated hydrogels proved to perform optimally.

We then exhaustively probed the sorption patterns of ferrous D-gluconate and its complexes with cyclodextrin derivatives. Through this investigation, we uncovered the roles played by both the hydrogel matrix and the particles themselves in mediating complexation events.

Given the paucity of the prior literature on cargo release kinetics from such composites, we performed meticulous evaluations of iron and cyclodextrin release dynamics from the hydrogel matrix under simulated digestive tract conditions. Notably, we observed a fascinating phenomenon: the iron D-gluconate complex releases promptly, while particles contribute to a temporally delayed release of a portion of the iron. Mathematical modeling indicated that the Higuchi model best describes iron release kinetics, whereas the CP model fits the release of cyclodextrin derivatives more appropriately.

Overall, these findings lay the groundwork for advancing the development of composite materials suitable for drug delivery applications, particularly in the realm of treating iron deficiency anemia.

## 4. Materials and Methods

### 4.1. Materials

The following chemicals were used: Sulfobutyl ether β-cyclodextrin sodium salt (SBECD, CAS 182410-00-0, Shanghai Macklin Biochemical Technology Co., Ltd., Shanghai, China); hexamethylene diisocyanate (HMD, Sigma-Aldrich, Darmstadt, Germany), dimethyl sulfoxide (DMSO, Sigma-Aldrich, Darmstadt, Germany); hydrochloric acid (35%, 36.46 g/mol, 1.18 g/cm^3^, purity ≥ 99%, Khimmed, Moscow, Russia); methyltriethoxysilane (Reakhim, Moscow, Russia); 3-aminopropyltriethoxysilane (ABCR, Karlsruhe, Germany); anhydrous sodium hydroxide (NaOH, 39.99 g/mol, purity ≥ 95%, Komponent Reaktiv, Moscow, Russia); Congo red dye, TRIS, KH_2_PO_4_, Na_2_HPO_4_ (Sigma-Aldrich, Saint Louis, MO, USA); ferrous D-gluconate dihydrate (Fe[HOCH_2_(CHOH)_4_CO_2_]_2_ × 2H_2_O, 99.7%, Ruschim, Moscow, Russia); sodium chloride (NaCl, 58.44 g/mol, purity 99.9%, Reakhim, Moscow, Russia); NH_4_Fe(SO_4_)_2_ × 12H_2_O, NH_2_OH × HCl, ortho-Phenanthroline × 12H_2_O, H_2_SO_4_, NH_4_OH (Reakhim, Moscow, Russia).

### 4.2. Methods

#### 4.2.1. Synthesis of PMSSO Hydrogels

Synthesis was conducted according to the methodics published previously [6,7], as illustrated in Scheme 3.

In brief, for synthesizing polymethylsilsesquioxane sol, an aqueous solution of sodium hydroxide (25.4 g, 0.634 mol) was added to a stirred mixture of methyltriethoxy-silane (112.9 g, 0.634 mol). After stirring for 30 min, a clear sol solution was formed. This process took place at ambient conditions.

PMSSO hydrogel without any cross-links was synthesized following the methodics in [14], where an acetic acid solution (24.1 g, 0.389 mol) in water (218 mL) was immediately added to a stirred polymethylsilsesquioxane sol (300 g). The gel underwent similar processing steps as above—aging followed by filtration and rinsing to achieve neutrality.

To synthetize PMSSO hydrogel-1 with minimal cross-linking, a sodium silicate sol (70.3 g, 0.575 mol) dissolved in water (68 mL) was mixed into a stirred polymethylsilsesquioxane sol (139 g). Acetic acid solution (25.2 g, 0.421 mol) in water (140 mL) was quickly introduced while stirring. Following this, the resulting gel was aged for 20 h before being washed with a small amount of 36.5% HCl until reaching pH 7. This process took place under ambient conditions.

The synthesis of PMSSO hydrogel-2 with denser cross-linking involved adding a sodium silicate sol (70.3 g, 0.575 mol) in water (68 mL) to a stirred polymethylsilsesquioxane sol (69.2 g). An acetic acid solution (13.2 g, 0.221 mol) in water (113 mL) was subsequently added swiftly during mixing. As previously described, after 20-hour aging, the product was filtered and rinsed with HCl solution until achieving pH 7.

For preparing amino-PMSSO, 3-aminopropyltriethoxysilane (48.7 g, 0.06 mol) was combined with methyltriethoxysilane (78.4 g, 0.114 mol), followed by the addition of water (62 mL) to the stirred mixture. Stirring continued for another 30 min, leading to the formation of a clear, viscous solution, which was also subjected to the aging and subsequent washing procedures mentioned earlier. All reactions were conducted at room temperature.

#### 4.2.2. Synthesis of the SBECD Derivatives

Schematic representation of the synthesis could be observed in Scheme 1. An aqueous solution of SBECD (20 mg/mL, 50 mL) was exposed at 40 °C and stirred (100 rpm) for 30 min until completely dissolved of cyclodextrin. Then, DMSO (48 or 46 mL) was added to the monomer solution under stirring (the water/DMSO ratio in the final solution was maintained constant and equal to 1:1 *v*/*v*). At the same time, 0.1 M cross-linking agent HMD solution in DMSO was prepared under stirring. After 10–15 s, an aliquot of 2 or 4 mL was taken from the obtained HMD solution and quickly added dropwise to the SBECD solution with constant stirring. After that, the solution was stirred for 2 h, and 500 µL concentrated hydrochloric acid was introduced.

The samples were purified from DMSO by dialysis using an MWCO Serva 14 kDa dialysis membrane. The systems were incubated for 24 h at 37 °C, with intense shaking and periodic renewal of the external aqueous solution, while monitoring the content of DMSO and SBECD by UV and IR spectra. Finally, the solutions were lyophilized.

#### 4.2.3. SBECD Oligomer—Ferrous Gluconate Complexes Formation

The schematic representation of the complex formation could be observed in Scheme 2. The dried oligomeric particles were distilled by adding 500 µL of water to the falcon tube containing them. Then, the suspension was stirred at 120 rpm for ~1 h at room temperature. Afterwards, the equivalent concentration of SBECD in the oligomers was defined.

Simultaneously, an aqueous solution of ferrous gluconate (0.1 M, 5 mL) was prepared and aliquoted into 200 µL portions. These portions were then diluted with distilled water and mixed with the oligomer solution to a final volume of 500 µL.

The oligomer solution was fractioned to achieve varying concentration ratios of SBECD to ferrous gluconate in the final solution. Only the initial volume of the ferrous gluconate and the final solution volume remained constant: 200 and 500 µL, respectively.

Finally, a series of samples was obtained, with equivalent concentrations of SBECD ranging from 0 to 0.05 M and a fixed ferrous gluconate concentration of 0.04 M. This series was analyzed by ATR-FTIR spectra to show the effectiveness of the sorption of free gluconate. It was determined by the reduction in the carboxyl group stretching peak intensity of free gluconate.

#### 4.2.4. Nanoparticle Tracking Analysis (NTA)

The nanoparticle tracking analysis (NTA) was conducted utilizing the NanoSight LM10-HS instrument manufactured by NanoSight Ltd. (Malvern, UK). Samples of SBECD cross-linked particles were diluted with Milli-Q purified water to achieve a concentration of approximately 100 particles per mL. Each sample underwent three replicate measurements, and the results are presented along with their respective standard deviations.

We determined Pol-SBECD molecular weight by the following formula according to the methodic published in [21]:

(1) $$M=\frac{[SBECD]\times N_{A}}{n}\times M_{SBECD}\;$$

The number of the particles *n* (particles/mL) was determined by NTA. Other variables are the concentration of SBECD torus [SBECD] (mole/mL) in accordance with the dilution, the Avogadro constant N_A_, and the molecular weight of the repeating unit (SBECD and linker) M_SBECD_.

#### 4.2.5. Dynamic Light Scattering (DLS)

DLS was used to determine the ζ-potentials of the samples (Zetasizer Nano S, Malvern, with 4 mW He–Ne-laser, 633 nm; Malvern, UK). The experiments were performed at 25 °C using the correlation of the K7032-09 Correlator System (Malvern, UK) and Zetasizer software v3.30. The measurements were carried out three times for each sample, and the values are reported with standard deviations.

#### 4.2.6. ATR-FTIR Spectroscopy

FTIR spectroscopic analyses were performed on a Bruker Tensor 27 spectrometer from Bruker (Ettlingen, Germany), which included a liquid nitrogen-cooled MCT detector, a temperature control unit supplied by Huber (Offenburg, Germany), and an attenuated total reflectance accessory with zinc selenide (ZnSe) single-reflection crystal. Spectra of the samples (volume 40–50 µL) were acquired in triplicate, consisting of 70 scans each, over the wavenumber range of 3000–900 cm^−1^ at a spectral resolution of 1 cm^−1^ maintained at 22 °C. A dry air stream was continuously circulated throughout the measurement process via an air compressor sourced from Jun-Air (Munich, Germany). Background readings (buffer solution) were collected following identical procedures. Data processing and interpretation were accomplished using the Opus 7.0 software package.

#### 4.2.7. Ortho-Phenanthroline Assay

The technique involves forming a colored complex between Fe^2+^ ions and ortho-phenanthroline, followed by quantification through UV-VIS absorbance measurements [30]. Initially, all forms of iron in the sample are reduced to the Fe^2+^ state using hydroxylamine as a reducing agent (prepared as a 0.1 g/mL aqueous solution).

During a routine experimental procedure, an aliquot of the iron-containing solution (up to 20 mL) is mixed with hydroxylamine hydrochloride solution (1 mL), acetate buffer solution (pH 4.5, 500 mM, 2 mL), and ortho-phenanthroline reagent (1.5 mL); this mixture is subsequently brought up to a final volume of 50 mL using distilled water. The resulting absorbance signal is measured spectrophotometrically at a wavelength of 510 nm. The concentration of Fe^2+^ was calculated by means of the calibration curve.

#### 4.2.8. N_2_ Adsorption–Desorption Isotherms

Isotherm data were obtained using a TMAX 3H-2000PM2 gas sorption analyzer produced by Xiamen Tmax Battery Equipments Limited (Xiamen City, China) at a temperature of −196 °C. Prior to analysis, all specimens underwent degassing treatment under vacuum conditions at 120 °C for a duration of 12 h. The specific surface area (SSA) according to the Brunauer–Emmett–Teller (BET) model was derived from the linear portion of the adsorption isotherm corresponding to relative pressures ranging from 0.05 to 0.3 P/P_0_.

#### 4.2.9. Scanning Electron Microscopy and EDX

Scanning electron microscopy (SEM) experiments were carried out employing an FEI Helios G4 CX dual-beam SEM instrument (Hillsboro, OR, USA), complemented by the SPI and TEAM 3D IQ 3.11 software packages. Before imaging, the samples were prepared by coating them with a thin silver (Ag) film (~10 nanometers thick) using equipment from SPI Supplies (West Chester, PA, USA).

Elemental distribution maps for iron (Fe) and silicon (Si) were obtained across an area of 150 square microns, utilizing the SEM-EDX analytical configuration enhanced with TEAM accessories.

#### 4.2.10. Fe Sorption by the Hydrogels

Ferrous D-gluconate solution (1 mL, 0.24 M) was added to 100 mg of hydrogel. Subsequently, the samples were incubated for 1.5 h at 37 °C with agitation at 200 rpm. Following incubation, the samples were centrifuged, and the iron content in the supernatant was determined using an ortho-phenanthroline assay.

#### 4.2.11. Determination of the Sorption Capacity of Hydrogels with Congo Red

A total of 50 mL of Congo red standard solution was added to 2.0 mL of hydrogel. This mixture was agitated for 30 min, followed by centrifugation at 6000 rpm for 20 min. Subsequently, 2 mL of the resultant supernatant fluid was transferred to a 50 mL volumetric flask. The volume was adjusted to the mark using a 0.02 M sodium chloride solution and thoroughly mixed. The absorbance of the solution was evaluated at its peak wavelength of 492 nm using UV spectroscopy, employing a quartz cuvette with a path length of 10 mm. For reference purposes, a 0.02 M sodium chloride solution served as the blank. Concurrently, the absorbance of the original Congo red solution was also assessed by UV spectroscopy.

The adsorption activity of hydrogel was calculated by the equation:

(2) $$X=\frac{(A_{0}-A)\times 5000}{A_{0}\times a\times M}$$

A_0_—optical density of the solution of Congo red; A—optical density of the solution of Congo red after sorption; a_0_—Congo red, g; a—hydrogel, g; M—Congo red molecular mass, g/mol.

Preparation of Congo red solution. Congo red (0.381 g, equivalent to 0.0005 moles) was initially weighed and placed inside a 1000-milliliter volumetric flask. To this, a 0.02 M sodium chloride solution (500 milliliters) was carefully added. Afterward, the contents were diluted to the full 1 L mark using additional 0.02 M NaCl solution before thorough mixing took place. Following that step, precisely 2 milliliters of the Congo red solution were pipetted into another volumetric flask capable of holding 50 milliliters. Finally, the volume was topped off to the 50-milliliter mark with further addition of 0.02 M sodium chloride solution, ensuring proper homogeneity through subsequent mixing.

#### 4.2.12. Complex SBECD Derivatives—Ferrous D-Gluconate Formation

Schematic representation of the synthesis could be observed in Scheme 2. A solution of ferrous D-gluconate + SBECD cross-linked particles or SBECD was prepared by mixing a 0.24 M salt solution and a particle solution (the particle concentration was determined by ATR-FTIR with 0.1 M) in certain proportions, so that the resulting solution had a SBECD torus/ferrous D-gluconate molar ratio of 1:1. This solution was incubated for an hour at 37 degrees Celsius and a stirring speed of 150 rpm.

#### 4.2.13. Hydrogels Loading Experiments

Schematic representation of the synthesis could be observed in Scheme 2. The solution obtained in Section 4.2.11 was poured into the gel and placed in an incubator for 1.5 h at 37 degrees Celsius and a stirring speed of 150 rpm. In the case of loading hydrogels with only ferrous D-gluconate or only SBECD particles, the volume of the other component was replaced by water. After this, it was centrifuged (10,000 rpm, 2 min for complete sedimentation of the gel particles), then the supernatant was collected, and, thus, saturated gels were obtained.

#### 4.2.14. Release Experiments

Release studies for each type of hydrogel were conducted over a cumulative period spanning up to five hours—two hours immersed in a simulated gastric environment succeeded by three hours in a simulated intestinal milieu. Throughout these tests, samples were kept at a constant temperature of 37 °C and 150 rpm. At every interval of twenty minutes, aliquots of the supernatant were withdrawn and immediately substituted with freshly prepared buffer solutions. The concentration of released iron ions was quantitatively assessed via the ortho-phenanthroline colorimetric method.

Gastric medium was prepared according to the Clark-Lubs’ methodology. This entailed dissolving 1.4635 g of sodium chloride (NaCl) in deionized water with 2.3 milliliters of concentrated hydrochloric acid (HCl). Final adjustment of the solution’s volume was achieved by filling a 500-milliliter volumetric flask with distilled water, yielding a standardized pH level of 1.4. The intestinal medium was prepared as follows. A buffered solution composed of 250 milliliters of Tris-HCl (125 milliliters of 0.1 Molar Tris base dissolved at a concentration of 12.114 g per liter), 105 milliliters of 0.1 Molar HCl, and completing the formulation with 20 milliliters of distilled water. Adjustment of the buffer’s final pH to 7.4 ensured physiological relevance.

Determination of the Fe content relied on the ortho-phenanthroline assay, whereas detection of the presence of cyclodextrins was conducted by monitoring the intensity of the characteristic absorption band located at 1040 cm^−1^ within the ATR-FTIR spectral area.

### 4.3. Mathematical Processing of the Data

Drug release models were considered as follows. The zero-order release model is described by Equation (3):

(3) $$Q_{t}\;={\;Q}_{\infty }\;+\;K_{0}t$$

where $K_{0}$ is the zero-order release constant, min^−1^; $Q_{t}$ is the amount of substance released at time t; $Q_{\infty }$ is the total amount of substance released.

The first-order release model corresponds to Equation (4):

(4) $$Q_{t}\;=\;Q_{\infty }\;\times \;e^{-K_{1}t}$$

where $K_{1}$ is the first-order release constant, min^−1^.

The Korsmeyer–Peppas release is described by Equation (5):

(5) $$\frac{Q_{t}}{Q_{\infty }}\;=\;K\;\times \;t^{n}$$

where *n* is the release index; K is the degree of release constant, min^−1^.

The Higuchi release model is represented by a complex relationship (6):

(6) $$Q_{t}\;=\;A\;\times \;\sqrt{\frac{D\;\times \;\varepsilon }{\tau }\;\times \;\left(2\;\times \;C-\varepsilon \;\times \;C_{s}\right)\;\times \;C_{s}\;\times \;t}\;=\;K_{H}\;\times \;\sqrt{t}\;$$

where $K_{H}$ is the Higuchi dissolution constant, min^−1/2^; D is the diffusion coefficient, sm^2^/min; C is the initial concentration of the released substance; τ is the tortuosity factor of the capillary system of the matrix (the value of the radius and branching of the channels and pores of the matrix); A is the unit of surface from which the amount of substance $Q_{t}$ during time t; $C_{s}$ is the solubility of the substance in the matrix; ε is the porosity of the matrix (the number of channels and pores).

### 4.4. Statistical Analysis

All experiments were performed in triplicate, and the results are presented as the mean value ± standard deviation (SD, *n* = 3). Statistical analysis was carried out using AtteStat 3.04 software for Microsoft Excel. The significance of differences between groups was assessed using the Mann–Whitney U-test, considering values with *p* ≤ 0.05 as statistically significant.

## Figures and Tables

**Table 1:** Key parameters of the synthetized SBECD cross-linked nanoparticles.

Sample	HMDI Molar Excess	Rh, nm (SD, *n* = 11)	ζ-Potential, mV (SD, *n* = 3)	Mr, kDa	Calculated Degree of Polymerization
SBECD (control)	0	0.15	−7.2 ± 0.8	2.1	-
Pol-SBECD-1	0.5	300 ± 20	−38.8 ± 1.2	210	100
Pol-SBECD-2	1	220 ± 25	−35.3 ± 0.6	154	73

**Table 2:** PMSSO hydrogels sorption capacity and specific surface area.

Sample Name	Composition	Sorption Capacity, µmol/g	Specific Surface Area, m^2^/g	Pore Volume, mL/g	Pore Diameter, nm
PMSSO hydrogel	Silsesquioxane structure only	4.5 ± 0.4	620 ± 10	2.007	2.253
Cross-linked PMSSO hydrogel 1	Silsesquioxane structure with 1:1 MeSiO_1.5_/SiO_2_ links molar ratio	1.0 ± 0.2	280 ± 5	0.38	4.8
Cross-linked PMSSO hydrogel 2	Silsesquioxane structure with 1:2 MeSiO_1.5_/SiO_2_ links molar ratio	0.2 ± 0.1	315 ± 6	0.61	6.6
Amino-PMSSO hydrogel	Contains amino groups (30% molar APTES)	84.9 ± 4.3	210 ± 8	0.36	6.1

**Table 3:** Fe loading efficacy for the samples under consideration.

Sample	Loading Efficacy, %
Amino-PMSSO@ferrous D-gluconate	11 ± 2
Amino-PMSSO@SBECD@ferrous D-gluconate	44 ± 1
Amino-PMSSO@Pol-SBECD-1@ferrous D-gluconate	26 ± 1
Amino-PMSSO@Pol-SBECD-2@ferrous D-gluconate	33 ± 1
PMSSO@ferrous D-gluconate	10 ± 1
PMSSO@SBECD@ferrous D-gluconate	19 ± 2
PMSSO@Pol-SBECD-1@ferrous D-gluconate	25 ± 1
PMSSO@Pol-SBECD-2@ferrous D-gluconate	13 ± 1
Cross-linked PMSSO-hydrogel 1@SBECD@ferrous D-gluconate	16 ± 2
Cross-linked PMSSO-hydrogel 2@SBECD@ferrous D-gluconate	14 ± 2

**Table 4:** R^2^ values obtained by mathematical processing of the initial section of the release curve from the system within the framework of several mathematical models of cyclodextrin tori release.

Sample/Model	0 Order	1st Order	Higuchi	Korsmeyer–Peppas
Amino-PMSSO@Pol-SBECD-1	0.9926	0.9586	0.9998	0.9968
Amino-PMSSO@Pol-SBECD-2	0.9928	0.9533	0.9963	0.9963
Amino-PMSSO@Pol-SBECD-1@ferrous D-gluconate	0.998	0.9459	0.9889	0.999
Amino-PMSSO@Pol-SBECD-2@ferrous D-gluconate	0.9904	0.9258	0.9913	0.9993
PMSSO@Pol-SBECD-1	0.9404	0.8718	0.9988	0.9992
PMSSO@Pol-SBECD-2	0.9397	0.8679	0.9985	0.9961
PMSSO@Pol-SBECD-1@ferrous D-gluconate	0.9659	0.8963	0.9997	0.9974
PMSSO@Pol-SBECD-2@ferrous D-gluconate	0.9145	0.8168	0.998	0.9892

**Table 5:** R^2^ values obtained by mathematical processing of the initial section of the release curve from the system within the framework of several mathematical models of ferrous D-gluconate release.

Sample/Model	0 Order	1st Order	Higuchi	Korsmeyer–Peppas
Amino-PMSSO@ferrous D-gluconate	0.8638	0.7524	0.9109	0.9967
Amino-PMSSO@Pol-SBECD-1@ferrous D-gluconate	0.7941	0.7059	0.9913	0.9608
Amino-PMSSO@Pol-SBECD-2@ferrous D-gluconate	0.7777	0.6918	0.9904	0.9574
PMSSO@ferrous D-gluconate	0.8706	0.8578	0.9661	0.9653
PMSSO@Pol-SBECD-1@ferrous D-gluconate	0.7213	0.6758	0.992	0.9803
PMSSO@Pol-SBECD-2@ferrous D-gluconate	0.6931	0.6419	0.9661	0.9163

## Data Availability

The original contributions presented in this study are included in the article/Supplementary Materials. Further inquiries can be directed to the corresponding author.

## Supplementary Materials

The following supporting information can be downloaded at https://www.mdpi.com/article/10.3390/gels11120973/s1. Figure S1: ATR-FTIR spectra of SBECD (A), cross-linked particles Pol-SBECD-1 (B, red line) and Pol-SBECD-2 (blue line), ferrous D-gluconate (C). Aqueous solutions at 22 °C. Figure S2: Scanning electron microscopy of cross-linked hydrogel PMSSO 1. Figure S3: Scanning electron microscopy of cross-linked hydrogel PMSSO 2. Figure S4: Scanning electron microscopy of amino-PMSSO hydrogel. Figure S5: Scanning electron microscopy of PMSSO hydrogel.

## References

[1] Iolascon A., Andolfo I., Russo R., Sanchez M., Busti F., Swinkels D., Aguilar P. (2024). Recommendations for diagnosis, treatment, and prevention of iron deficiency and iron deficiency anemia. Hemasphere.

[2] Ciont C., Pop R.M., Pop L., Vodnar D.C., Morariu I.D., Suharoschi R., Pop O.L. (2025). Impact of Side Effects on Anemia Therapy Compliance. Nutrients.

[3] Rajapaksha W., Nicholas I.H.W., Thoradeniya T., Karunaratne D.N., Karunaratne V. (2024). Novel alginate nanoparticles for the simultaneous delivery of iron and folate: A potential nano-drug delivery system for anaemic patients. RSC Pharm..

[4] Szostak-Paluch K., Drabik D., Jędruchniewicz N., Dwornikowska-Dąbrowska M. (2024). In vitro studies of a novel liposomal formulation for safe and efficient iron delivery. Eur. J. Lipid Sci. Technol..

[5] Surekha B., Misra P., Thippaiah A.C., Shamanna B.R., Madathil A., Rajadurai M. (2024). A microneedle transdermal patch loaded with iron(ii) nanoparticles for non-invasive sustained delivery to combat anemia. Mater. Adv..

[6] Orlova P., Meshkov I., Sharikov S., Frolov V., Skuredina A., Markov P., Bobyleva Z., Lakienko G., Latipov E., Kolmogorov I. (2025). Amidated and Aminated PMSSO-Hydrogels as a Promising Enzyme-Sensitive Vehicle for Antianemic Drugs. Gels.

[7] Orlova P., Meshkov I., Latipov E., Vasiliev S., Mikheev I., Ratova D.M., Kalinina A., Muzafarov A., Le-Deygen I. (2024). Cyclodextrin—Polymethylsilsesquioxane Combined System as a Perspective Iron Delivery System for Oral Administration. Gels.

[8] Migulin D.A., Rozanova J.V., Meshkov I.B., Milenin S.A., Shitikov E.A., Muzafarov A.M. (2024). Functional Polyorganosilsesquioxane Nanocomposite Hydrogels With Encapsulated Silver Nanoparticles—Promising Compounds Against Gastrointestinal Pathogen Bacteria. ChemistrySelect.

[9] Wehl L., Muggli K., Möller K., Engelke H., Bein T. (2025). Cross-Linked Cyclodextrin-Based Nanoparticles as Drug Delivery Vehicles: Synthesis Strategy and Degradation Studies. ACS Omega.

[10] Demirci S., Khiev D., Can M., Sahiner M., Biswal M.R., Ayyala R.S., Sahiner N. (2021). Chemically Cross-Linked Poly(β-Cyclodextrin) Particles as Promising Drug Delivery Materials. ACS Appl. Polym. Mater..

[11] Skuredina A.A., Tychinina A.S., Le-Deygen I.M., Golyshev S.A., Kopnova T.Y., Le N.T., Belogurova N.G., Kudryashova E.V. (2022). Cyclodextrins and Their Polymers Affect the Lipid Membrane Permeability and Increase Levofloxacin’s Antibacterial Activity In Vitro. Polymers.

[12] Quaglia F., Varricchio G., Miro A., Immacolata La Rotonda M., Larobina D., Mensitieri G. (2001). Modulation of drug release from hydrogels by using cyclodextrins: The case of nicardipine/β-cyclodextrin system in crosslinked polyethylenglycol. J. Control. Release.

[13] Omidian H., Akhzarmehr A., Gill E.J. (2025). Cyclodextrin–Hydrogel Hybrids in Advanced Drug Delivery. Gels.

[14] Malik N.S., Ahmad M., Minhas M.U. (2017). Cross-linked β-cyclodextrin and carboxymethyl cellulose hydrogels for controlled drug delivery of acyclovir. PLoS ONE.

[15] Lin J., Chen Y., Wang X. (2025). Cyclodextrin-Based Supramolecular Hydrogels in Tissue Engineering and Regenerative Medicine. Molecules.

[16] Szente L., Singhal A., Domokos A., Song B. (2018). Cyclodextrins: Assessing the impact of cavity size, occupancy, and substitutions on cytotoxicity and cholesterol homeostasis. Molecules.

[17] Liu Y., Lin T., Cheng C., Wang Q., Lin S., Liu C., Han X. (2021). Research progress on synthesis and application of cyclodextrin polymers. Molecules.

[18] Gabr M.M., Mortada S.M., Sallam M.A. (2018). Carboxylate cross-linked cyclodextrin: A nanoporous scaffold for enhancement of rosuvastatin oral bioavailability. Eur. J. Pharm. Sci..

[19] Cakić S.M., Ristić I.S., Holló B.B., Nikolić V., Nikolić N., Rakić S., Ilić-Stojanović S. (2024). Thermoanalytical studies on cross-linked polyurethane networks: Effect of polyol molecular weight and structure of cyclodextrins. Polym. Bull..

[20] Chen H., Xu H., Zhang Y., Zhou J., He J., Wang W., Yuan C., Zhao C., Yang L. (2023). A double-crosslinked cyclodextrin-based porous polymer for effective removal of bisphenol A: Preparation, adsorption behavior and mechanism. J. Environ. Chem. Eng..

[21] Skuredina A.A., Tychinina A.S., Le-Deygen I.M., Golyshev S.A., Belogurova N.G., Kudryashova E.V. (2021). The formation of quasi-regular polymeric network of cross-linked sulfobutyl ether derivative of β-cyclodextrin synthesized with moxifloxacin as a template. React. Funct. Polym..

[22] Meshkov I.B., Mazhorova N.G., Bakirov A.V., Vasil’ev S.G., Kalinina A.A., Bystrova A.V., Muzafarov A.M. (2025). Evolution of Methylsilsesquioxane: From Hydrolytic Polycondensation Product to Xerogel. Polymers.

[23] Luan V.H., Tien H.N., Hoa L.T., Hien N.T.M., Oh E.S., Chung J., Kim E.J., Choi W.M., Kong B.S., Hur S.H. (2013). Synthesis of a highly conductive and large surface area graphene oxide hydrogel and its use in a supercapacitor. J. Mater. Chem. A.

[24] Kass L.E., Nguyen J. (2022). Nanocarrier-hydrogel composite delivery systems for precision drug release. Wiley Interdiscip. Rev. Nanomed. Nanobiotechnol..

[25] Trucillo P. (2022). Drug Carriers: A Review on the Most Used Mathematical Models for Drug Release. Processes.

[26] Katzhendler I., Hoffman A., Goldberger A., Friedman M. (1997). Modeling of Drug Release from Erodible Tablets. J. Pharm. Sci..

[27] Bayer I.S. (2023). Controlled Drug Release from Nanoengineered Polysaccharides. Pharmaceutics.

[28] Khobragade P.P., Manvatkar V., Meshram P., Dongre R. (2024). Multi-Purpose Cyclodextrin Metal-Complexes: Physicochemical and Theoretical Portfolio in Drug Domain. J. Chem. Health Risks.

[29] MacHín R., Isasi J.R., Vélaz I. (2012). β-Cyclodextrin hydrogels as potential drug delivery systems. Carbohydr. Polym..

[30] Smith L.E. (1938). Analysis of Commercial Phenothiazine Used as an Insecticide. Ind. Eng. Chem.-Anal. Ed..

