# Supplementary material for: Advanced PMSSO Hydrogel Cross-Linked Cyclodextrin Composite Carrier for Enhanced Oral Delivery of Iron to Treat Anemia
Source: Gels. 2025 Dec 2;11(12):973. doi: 10.3390/gels11120973 (PMC12732416; doi:10.3390/gels11120973)
Supplement: Supplementary file 1 [file gels-11-00973-s001.zip › gels-3980711-supplementary.pdf]

Supplementary Materials

Advanced PMSSO Hydrogel Cross-Linked Cyclodextrin Composite Carrier for Enhanced Oral Delivery of Iron to Treat Anemia

Polina Orlova [1], Sergei Sharikov [1], Vsevolod Frolov [1], Alexey Doroshenko [1], Ivan Meshkov [2], Anna Skuredina [1], Grigorii Lakienko [1], Egor Latipov [3], Alexandra Kalinina [2], Aziz Muzafarov [2] and Irina Le-Deygen [1,*]

[1] Department of Chemistry, Lomonosov Moscow State University, Moscow 119991, Russia
[2] Enikolopov Institute of Synthetic Polymeric Materials, Russian Academy of Sciences (ISPM RAS), Moscow 117393, Russia
[3] Institute of Nanotechnology of Microelectronics, Russian Academy of Sciences (INME RAS), Moscow 115487, Russia
* Correspondence: i.m.deygen@gmail.com

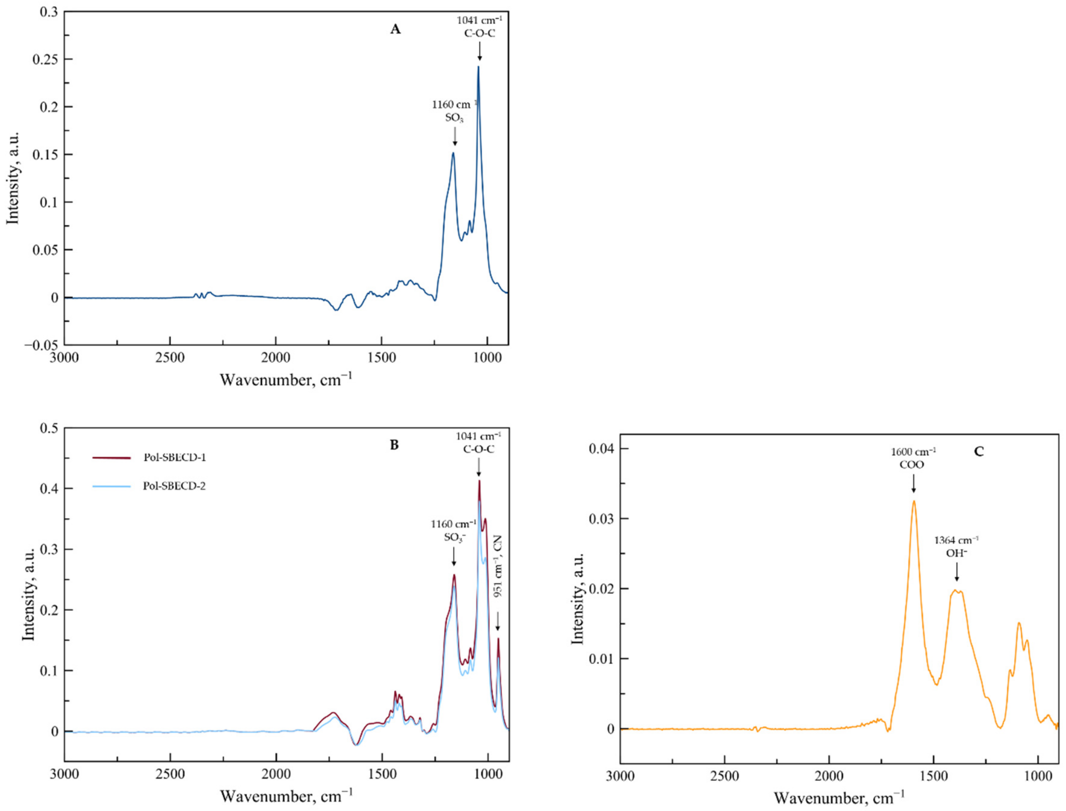

Figure S1. ATR-FTIR spectra of SBECD (A), cross-linked particles Pol-SBECD-1 (B, red line) and Pol-SBECD-2 (blue line), ferrous D-gluconate (C). Aqueous solutions at 22°C.

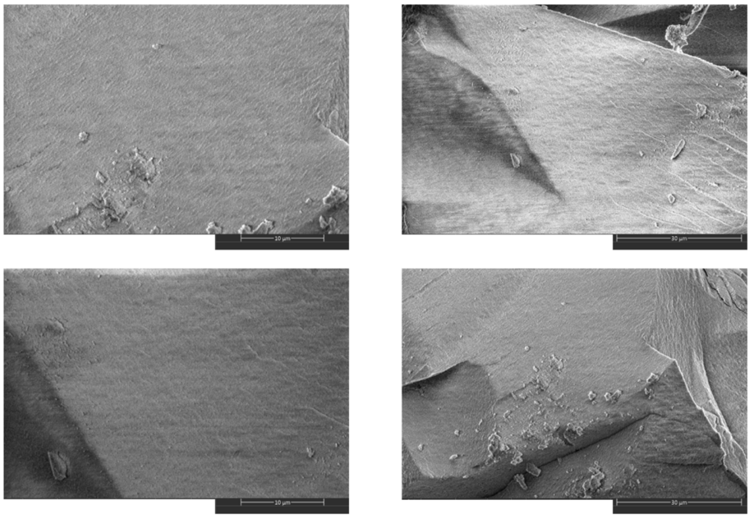

Figure S2. Scanning electron microscopy of cross-linked hydrogel PMSSO 1.

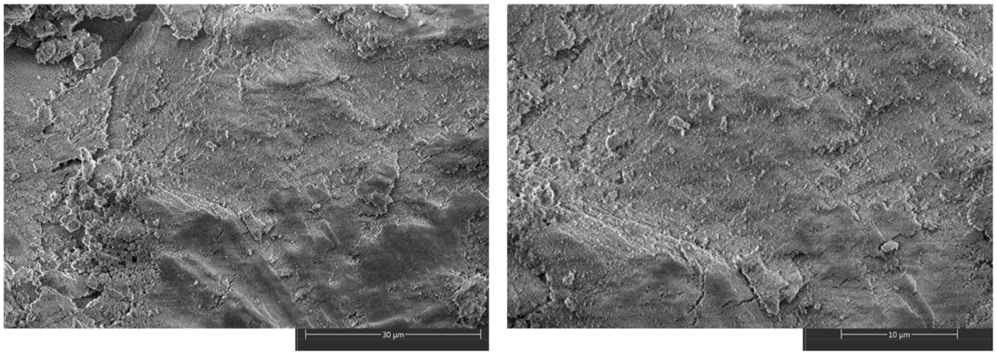

Figure S3. Scanning electron microscopy of cross-linked hydrogel PMSSO 2.

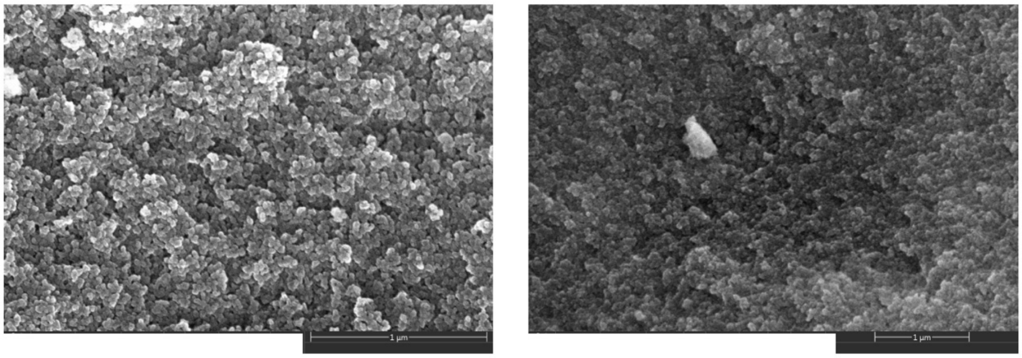

Figure S4. Scanning electron microscopy of Amino-PMSSO hydrogel.

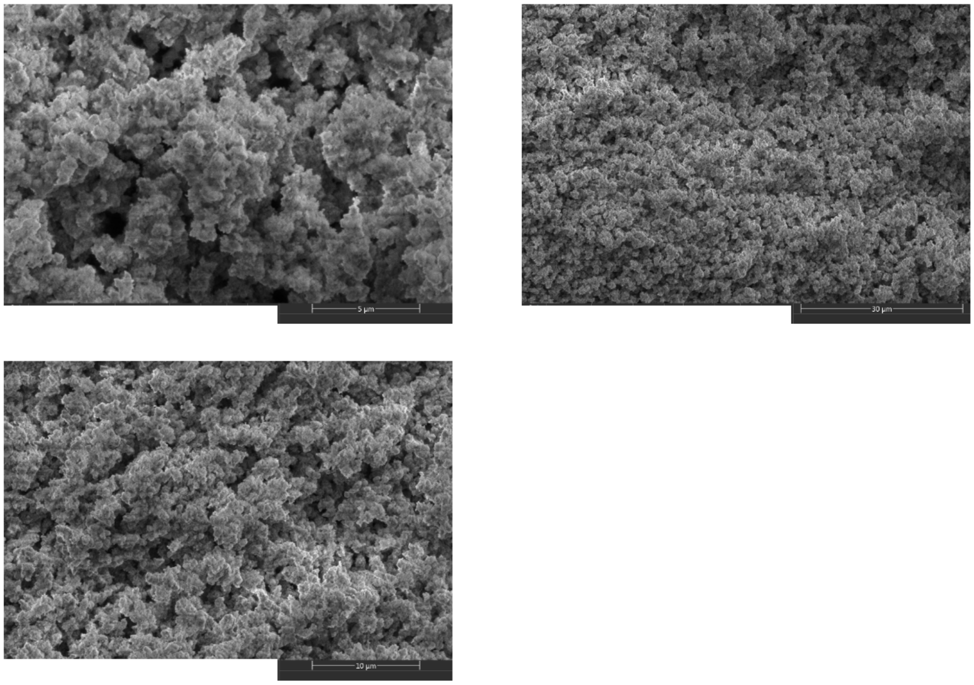

Figure S5. Scanning electron microscopy of PMSSO hydrogel.
